# *In Vitro* Properties of Potential Probiotic Indigenous Lactic Acid Bacteria Originating from Traditional Pickles

**DOI:** 10.1155/2015/315819

**Published:** 2015-05-26

**Authors:** Mehmet Tokatlı, Gökşen Gülgör, Simel Bağder Elmacı, Nurdan Arslankoz İşleyen, Filiz Özçelik

**Affiliations:** ^1^Department of Food Engineering, Faculty of Natural Sciences and Engineering, Gaziosmanpaşa University, 60150 Tokat, Turkey; ^2^Department of Food Engineering, Faculty of Agriculture, Uludağ University, 16059 Bursa, Turkey; ^3^Department of Food Engineering, Faculty of Engineering, Ankara University, 06110 Ankara, Turkey; ^4^Yeniçağa Yaşar Çelik Vocational School, Abant İzzet Baysal University, 14650 Bolu, Turkey

## Abstract

The suitable properties of potential probiotic lactic acid bacteria (LAB) strains (preselected among 153 strains on the basis of their potential technological properties) isolated from traditional Çubuk pickles were examined *in vitro*. For this purpose, these strains (21 *Lactobacillus plantarum*, 11 *Pediococcus ethanolidurans,* and 7 *Lactobacillus brevis*) were tested for the ability to survive at pH 2.5, resistance to bile salts, viability in the presence of pepsin-pancreatin, ability to deconjugate bile salts, cholesterol assimilation, and surface hydrophobicity properties. Most of the properties tested could be assumed to be strain-dependent. However, *L. plantarum* and *L. brevis* species were found to possess desirable probiotic properties to a greater extent compared to *P. ethanolidurans*. In contrast to *P. ethanolidurans* strains, the tested *L. plantarum* and *L. brevis* strains exhibited bile salt tolerance, albeit to different extent. All tested strains showed less resistance to intestinal conditions than gastric juice environment. Based on the survival under gastrointestinal conditions, 22 of the 39 strains were selected for further characterization. The eight strains having the highest cholesterol assimilation and surface hydrophobicity ratios could be taken as promising probiotic candidates for further *in vivo* studies, because of the strongest variations found among the tested strains with regard to these properties.

## 1. Introduction

There has been an increasing interest in functional foods with health-promoting attributes. Within this context, probiotic foods have received considerable attention in recent years [1]. Probiotics are defined as “live microorganisms that, when administered in adequate amounts, confer a health benefit on the host” [2], as updated by Hill et al. [3]. The beneficial health effects claimed for probiotics are regulation of microbial balance in the gastrointestinal tract, reduction of serum cholesterol levels, alleviation of lactose intolerance symptoms, lowering the risk of colon cancer, enhancement of nutrients bioavailability, prevention or reduction of the prevalence of allergies in susceptible individuals, enhancement of the immune system, and improvement of calcium absorption [1, 4–6]. As established by the Food and Agriculture Organization and the World Health Organization (FAO/WHO), the main currently used* in vitro* tests for the study of probiotic strains are resistance to gastric acidity, bile acid resistance, adherence to mucus and/or human epithelial cells and cell lines, antimicrobial activity against potentially pathogenic bacteria, ability to reduce pathogen adhesion to surfaces, and bile salt hydrolase activity [2].

The most commonly used probiotic microorganisms include various species of genera* Lactobacillus* and* Bifidobacterium*, as well as some* Bacillus*,* Streptococcus*,* Pediococcus*, and* Enterococcus* species [7, 8]. In the past, human/animal gastrointestinal tract was considered as the principal source of probiotic strains since those strains of host origin would be better adapted to colonize the human/animal gastrointestinal tract [9, 10]. Recently, fermented foods, in which probiotics are intended to be used, have drawn attention as source of probiotic organisms [9]. Dairy products have been considered as the best matrices to deliver probiotics [1, 11]. On the other hand, there is a growing interest in the development of non-dairy-based probiotic products due to the drawbacks related to the consumption of dairy products, including lactose intolerance and the unfavourable cholesterol content [4, 8]. Although the use of fermented fruit and vegetable products as raw material for probiotic microorganisms has started to be investigated in several studies [1, 4, 9], they are still scarce compared with dairy products. In this context, pickle which is a traditional fermented vegetable product could be a promising source of probiotic microorganisms.

The aim of this work was to study some suitable properties of potential probiotic LAB associated with pickles. Thirty-nine LAB isolates originating from naturally fermented pickles were subjected to* in vitro* analyses to determine their probiotic potential. The properties tested in this study include ability to survive at pH 2.5, resistance to bile salts (0.3% oxgall), viability in the presence of pepsin-pancreatin, ability to deconjugate bile salts, cholesterol assimilation, and surface hydrophobicity properties.

## 2. Materials and Methods

### 2.1. Bacterial Strains and Growth Conditions

A total of 39 indigenous LAB strains, isolated from pickles produced in Ankara-Çubuk region, were screened for their potential probiotic properties. The tested strains (preselected among 153 LAB strains on the basis of their potential technological properties, including growth ability in MRS Broth, acid production, and tolerance to low pH) included 21* L. plantarum*, 11* P. ethanolidurans,* and 7* L. brevis* strains which were previously identified by molecular methods. The GenBank accession numbers for the 16S rRNA gene sequences of the strains were reported previously [12]. The LAB strains were cultured at 30°C for 48 h in MRS Broth and/or MRS Agar as basal media.

### 2.2. Screening for Probiotic Properties

#### 2.2.1. Resistance to Low pH, Bile Salts, and Simulated Gastric and Intestinal Fluids

To determine the acid tolerance of strains, LAB cells were harvested by centrifugation at 6000 g for 15 min after incubation at 30°C for 48 h. The collected pellets were suspended in sterile PBS (phosphate-saline buffer; 9 g/L NaCl, 9 g/L Na_2_HPO_4_·2H_2_O, 1.5 g/L KH_2_PO_4_) adjusted to pH 2.5 to the initial volume. The mixture was then incubated at 37°C for 4 h. Aliquots of samples were taken at time 0 and after 4 h. These samples were serially diluted in sterile saline solution (0.85% NaCl) and the viable cell population was determined by the spread plate method using MRS Agar. The plates were incubated at 37°C for 48 h [13]. The percentage survival of the bacteria was calculated as follows:

(1) %survival=log  cfu  of  viable  cells  survivedlog  cfu  of  initial  viable  cells  inoculated×100.

For the bile salt tolerance assay, MRS Broth containing 0.3% (w/v) bile salt (oxgall) was inoculated with active LAB cultures (incubated at 30°C for 48 h) at an inoculum size of 1% (v/v) and incubated at 37°C for 4 h. The viable cell population was determined at 0 h and 4 h of incubation on MRS Agar plates by the spread plate method. The percentage survival of the bacteria was calculated according to (1) [14].

To test the viability in the presence of pepsin, simulated gastric juice which was prepared by suspending 3 mg/mL pepsin in sterile saline solution (0.85% NaCl, w/v) adjusted to pH 2.5 was inoculated with active LAB cultures (incubated at 30°C for 48 h) at an inoculum size of 1% (v/v) and incubated at 37°C for 4 h. Simulated intestinal fluid which was prepared by dissolving bile salt (0.3%) and pancreatin (1 mg/mL) in sterile saline solution (0.85% NaCl, w/v) adjusted to pH 8.0 was used in pancreatin resistance test. This fluid was inoculated with active LAB cultures at an inoculum size of 1% (v/v) and incubated at 37°C for 6 h. The viable cell population was determined before and after incubation on MRS Agar plates by the spread plate method. The percentage survival of the bacteria was calculated according to (1) [7, 15].

#### 2.2.2. Deconjugation of Bile Salts

Deconjugation of bile salt by LAB strains was tested through the plate assay as described by Ahn et al. [16]. 1 mM of sodium taurodeoxycholate hydrate (TDCA), taurocholic acid sodium salt hydrate (TCA), sodium taurolithocholate (TLCA), sodium glycocholate hydrate (GCA), and sodium taurochenodeoxycholate (TCDCA) were added either individually or as a mixture to MRS Agar to prepare Bile salt-MRS Agar plates. The plates were then inoculated with 10 *μ*L of active LAB cultures and incubated at 37°C for 72 h. Subsequently, diameters of the precipitate halos around colonies were measured.

#### 2.2.3. Cholesterol Assimilation

MRS Broth supplemented with 50 *μ*g/mL water-soluble form of cholesterol (PEG600, Sigma) was inoculated with active LAB cultures at an inoculum size of 1% (v/v). After incubation at 37°C for 24 h, the culture cells were removed by centrifugation. The collected supernatant and the control, which was the uninoculated sterile broth, were then assayed for their cholesterol content by OPA (o-phthalaldehyde) method as described by Rudel and Morris [17] with slight modifications by Gilliland et al. [18]. Differences in the cholesterol content between the control and the culture test tubes were taken as the assimilated amount of cholesterol.

#### 2.2.4. Surface Hydrophobicity

The adhesion ability of the organisms to hydrocarbons is used as a measure of their hydrophobicity. Briefly, LAB cells were harvested by centrifugation at 6000 g for 10 min, washed twice in 50 mM K_2_HPO_4_, and then resuspended in the same buffer to obtain an *A*_560 nm_ value of approximately 1.0. Three mL of bacterial suspension was put in contact with 0.6 mL of n-hexadecane by vortexing for 2 min. The phases were allowed to separate by decantation at 37°C for 1 h. The aqueous phase was carefully removed, and the *A*_560_ was measured. The decrease in the absorbance of the aqueous phase was taken as a measure of the cell surface hydrophobicity (*H*%), which was calculated with the formula *H*% = [(*A*_*o*_ − *A*)/*A*_*o*_]*∗*100, where *A*_*o*_ and *A* are the absorbance before and after extraction with n-hexadecane [19].

#### 2.2.5. Statistical Analyses

All experiments were conducted in two biological replicates, each with two technical replicates. Experimental data were analysed with one-way ANOVA using the Minitab statistical software, version 14 (Minitab Inc., State College, PA, USA). Statistical differences among means were determined by the Duncan's multiple range tests at the 5% significance level.

## 3. Results and Discussion

### 3.1. Resistance to Low pH, Bile Salts, and Simulated Gastric and Intestinal Fluids

Resistance to stomach pH, bile salts, and pancreatic fluid is of great importance in predicting the survival and growth of the potential probiotic strains in the gastrointestinal conditions [7, 15]. Tolerance to gastric acidity (pH 2.0–2.5) is considered as a key functional requirement for probiotics, which enables them to survive during passage through the gastrointestinal tract [20]. The viable counts of the 14 LAB strains were found to be below 1 log cfu/mL after 4 h of exposure to pH 2.5, and consequently these strains were not further tested. For the remaining 25 strains belonging to* L. plantarum*,* L. brevis,* and* P. ethanolidurans*, survival at pH 2.5 for 4 h was 35–85%, 33–64%, and 40–76%, respectively (Table 1). At the species level, the majority of* L. plantarum* and* L. brevis* strains showed higher resistance to low pH in comparison with* P. ethanolidurans* strains. The survival ability of lactobacilli at pH 2.5 was also reported by others [1, 21]. However, variability in acidic response was obtained among the tested strains (*p* < 0.05), indicating that resistance to low pH is a strain-specific property. Similarly, Jacobsen et al. [22] reported strain-dependent survival of* Lactobacillus* spp. isolated from Ghanaian fermented maize.

Resistance to bile salts is one of the most important selection criteria for probiotics since the small intestine and colon contain relatively high concentrations of bile salts which are toxic for living cells [5, 23]. It was reported that the different species of* Lactobacillus* showed significant variations in relation to their bile salt tolerance [24]. In this study, all studied* L. plantarum* and* L. brevis* strains showed varying levels of resistance to bile salts after 4 h of exposure whereas none of the examined* P. ethanolidurans* strains were able to withstand bile concentration of 0.3% (Table 2). In particular, 5 selected* L. brevis* strains were found to be highly tolerant to 0.3% oxgall exhibiting negligible reduction in viable counts (<1 log cycle) after 4 h of incubation (statistical groups labeled with the capital letters A, B, and AB in Table 2). These results were in accordance with the previous studies that showed that the lactobacilli possessed high tolerance to bile salts at 0.3% [5, 22]. In contrast, Sukumar and Ghosh [25] reported that* Pediococcus* spp. which were isolated from an Indian fermented food showed significant bile tolerance. The different survival rates for* L. plantarum* strains suggest that survival ability in the bile media is strain-dependent, as shown in previous reports [26, 27].

The combined effect of pepsin-pH 2.5 solution and pancreatin-bile salt solution (pH 8.0) aims at simulating the gastric juice and the intestine, respectively. The survival rates of LAB in simulated gastric and intestinal conditions are presented in Table 3.* Lactobacillus plantarum* and* L. brevis* strains had high survival rates in simulated gastric conditions except for* L. brevis* MF343, which could not survive after treatment by gastric juice. Although all studied* L. plantarum* and* L. brevis* strains could survive well in the presence of 0.3% oxgall alone, some of these strains were not able to withstand the simulated intestinal conditions since no viability was observed after 4 h of exposure. Therefore, it could be suggested that the decrease of viability was due to the pancreatin alone, or in synergy with bile salts.* Pediococcus ethanolidurans* strains were sensitive to both simulated gastric and intestinal conditions and were then discarded from further analysis. In general, all tested strains showed less resistance to intestinal conditions than gastric juice environment. This could be related to natural adaptation of LAB strains to low pH conditions since they are of pickle origin. Similarly, Grimoud et al. [28] reported that* Lactobacillus* strains had higher survival rates under simulated gastric conditions compared to intestinal conditions.

### 3.2. Deconjugation of Bile Salts

None of the tested strains had the ability to deconjugate bile salts since the cultures did not form precipitate halos around the colonies on Bile salt-MRS Agar plates (data not shown). The absence of BSH (bile salt hydrolysis) activity in LAB strains isolated from table olives was also reported in some previous studies [9, 29]. However, it is not straightforward to interpret this result since it is uncertain whether BSH activity is a desirable trait for probiotics because excessive amounts of deconjugated bile salts may be potentially detrimental to the human host [9, 29].

### 3.3. Cholesterol Assimilation

High concentration of cholesterol in the blood streams of humans is generally recognized as a risk factor for coronary heart disease [24, 30]. Consumption of fermented milk products containing certain lactobacilli or bifidobacteria has been asserted to reduce serum cholesterol levels in humans [16, 24, 30]. In this study, the cholesterol assimilation ratios varied depending on the tested strains and ranged from 0.83 to 48.56%.* Lactobacillus plantarum* strains MF556, MF143, MF169, and MF33 (statistical groups labeled with the capital letters A and B) exhibited significantly higher amounts of cholesterol assimilation compared to other strains tested. As shown in Table 4, these four strains were found to reduce the cholesterol level by 48.56, 47.69, 44.26, and 43.57%* in vitro* tests, respectively, corresponding to the amount of cholesterol assimilation at ca. 20 *μ*g/mL. The wide range of results has been reported on cholesterol assimilation levels of* Lactobacillus* spp. in previous studies: 13–38% [31], 14–22 *μ*g/mL [30], and 56–62 *μ*g/mL [24]. Cholesterol assimilation property appears to show variability across the species and strains. In addition, it is somewhat difficult to compare the present results with other reports since different initial concentration of cholesterol were used. Cholesterol lowering effect of probiotic bacteria has been explained by different proposed mechanisms which include bile salt hydrolase activity, production of compounds that inhibit enzymes such as 3-hydroxy-3-methylglutaryl coenzyme A, and cholesterol assimilation [31]. In the present study, it could be suggested that cholesterol lowering effect of the tested strains was not related to their deconjugation ability of bile salts, in contrast with that found by other authors [16]. On the other hand, Walker and Gilliland [32] found no significant correlation between the ability of* L. acidophilus* to deconjugate bile acids and the ability to assimilate cholesterol.

### 3.4. Surface Hydrophobicity

The colonization in intestinal wall is considered as a desirable property of probiotic bacteria [33]. In this context, the adhesion ability to the intestinal epithelium, which is considered to be a prerequisite for colonization, is an important criterion for the selection of probiotic bacteria [34]. The surface properties, like autoaggregation and hydrophobicity, are used as a measurement directly related to ability to adhere to cell monolayers [29, 33]. Some authors found a correlation between hydrophobicity and adhesion ability [35], while some others reported that hydrophobicity values do not correlate with adhesion properties [27]. In the present study, significant differences (*p* < 0.05) in hydrophobicity values were found between LAB strains, even within the same species. At the species level, the hydrophobicity values found ranged from 0.66 to 82.41% (*L. plantarum*) and from 0.17 to 97.96% (*L. brevis*) (Table 4). The results revealed that the greatest hydrophobicity for n-hexadecane was observed for* L. brevis* MF105,* L. plantarum* MF265,* L. brevis* MF494, and* L. brevis* MF493 at 97.96%, 82.41%, 67.29%, and 62.36%, respectively. These four isolates may be considered potential probiotic cultures, from the adhesive point of view. Kumar et al. [36] reported that four* Lactobacillus* spp., isolated from indigenous pickled vegetables and fermented beverages, exhibited high cell surface hydrophobicity (>60%).

## 4. Conclusion

Although most of the characteristics tested could be assumed to be strain-dependent,* L. plantarum* and* L. brevis* species were found to possess desirable* in vitro* properties to a greater extent compared to* P. ethanolidurans*. It is difficult to find a strain having all desirable functional properties, and the selection criteria for potential probiotic candidates could therefore be dependent on the purpose of the product. For instance,* L. plantarum* strains MF556, MF143, MF169, and MF33 exhibited the highest* in vitro* cholesterol assimilation among the tested strains whereas the maximum hydrophobicity for n-hexadecane was observed for* L. brevis* MF105,* L. plantarum* MF265,* L. brevis* MF494, and* L. brevis* MF493. Since the strongest variation was found among the tested 22 strains with regard to their cholesterol assimilation and surface hydrophobicity properties, these eight strains could be selected as promising probiotic candidates for further investigation* in vivo* studies to evaluate their potential health benefits and their application in the food industry.

## Figures and Tables

**Table 1:** Acid tolerance of LAB strains in PBS (pH 2.5).

Species	Strain number	Initial counts (log cfu/mL)	Survival after 4 h at pH 2.5	
			(log cfu/mL)	%
*L. plantarum *	MF303	9.47 ± 0.04	8.08 ± 0.16	85^A^
	MF169	9.22 ± 0.05	7.05 ± 0.17	76^B^
	MF4	8.54 ± 0.07	6.14 ± 0.07	72^C^
	MF213	9.45 ± 0.05	6.79 ± 0.10	72^C^
	MF143	9.28 ± 0.01	6.49 ± 0.01	70^CD^
	MF556	9.85 ± 0.00	6.60 ± 0.00	67^DE^
	MF376	9.94 ± 0.02	6.28 ± 0.05	63^FG^
	MF265	8.58 ± 0.06	5.16 ± 0.11	60^GH^
	MF548	9.65 ± 0.07	5.65 ± 0.16	59^H^
	MF380	9.68 ± 0.05	5.54 ± 0.09	57^H^
	MF239	9.37 ± 0.10	4.68 ± 0.07	50^I^
	MF33	9.25 ± 0.04	4.66 ± 0.19	50^I^
	MF178	8.96 ± 0.08	3.96 ± 0.03	44^J^
	MF352	9.21 ± 0.14	3.74 ± 0.26	41^JK^
	MF377	10.04 ± 0.05	4.06 ± 0.07	40^KL^
	MF205	9.45 ± 0.11	3.49 ± 0.02	37^LM^
	MF305	9.17 ± 0.07	3.18 ± 0.31	35^MN^
	MF150	9.23 ± 0.05	<1.00	—
	MF219	9.01 ± 0.11	<1.00	—
	MF357	9.83 ± 0.06	<1.00	—
	MF513	7.75 ± 0.11	<1.00	—

*L. brevis *	MF493	9.37 ± 0.09	5.99 ± 0.21	64^EF^
	MF105	9.22 ± 0.08	3.90 ± 0.12	42^JK^
	MF494	9.54 ± 0.12	3.96 ± 0.33	42^JK^
	MF343	8.80 ± 0.01	3.45 ± 0.01	39^KL^
	MF314	9.41 ± 0.12	3.13 ± 0.02	33^N^
	MF158	9.44 ± 0.04	<1.00	—
	MF354	9.10 ± 0.08	<1.00	—

*P. ethanolidurans *	MF179	9.54 ± 0.15	7.23 ± 0.06	76^B^
	MF180	9.79 ± 0.01	7.09 ± 0.04	72^C^
	MF50	9.45 ± 0.04	3.79 ± 0.05	40^KL^
	MF48	9.15 ± 0.11	<1.00	—
	MF107	9.01 ± 0.01	<1.00	—
	MF167	9.02 ± 0.01	<1.00	—
	MF194	9.16 ± 0.19	<1.00	—
	MF196	9.37 ± 0.14	<1.00	—
	MF214	9.41 ± 0.04	<1.00	—
	MF251	9.19 ± 0.07	<1.00	—
	MF269	9.21 ± 0.07	<1.00	—

Values are expressed in mean ± standard error.

^A–N^Values with different letters within a column indicate significant differences between LAB strains (*p* < 0.05).

**Table 2:** Bile salt tolerance of LAB strains.

Species	Strain number	Initial counts (log cfu/mL)	Survival after 4 h in the presence of 0.3% (w/v) bile salt (oxgall)	
			(log cfu/mL)	%
*L. plantarum *	MF213	9.52 ± 0.07	9.44 ± 0.07	99^A^
	MF205	8.22 ± 0.10	7.79 ± 0.12	95^B^
	MF377	8.91 ± 0.00	8.41 ± 0.02	94^B^
	MF239	7.68 ± 0.10	6.59 ± 0.16	86^C^
	MF143	8.14 ± 0.07	6.84 ± 0.09	84^CD^
	MF305	9.32 ± 0.07	7.51 ± 0.09	81^DE^
	MF169	8.45 ± 0.16	6.69 ± 0.30	79^EF^
	MF178	9.45 ± 0.04	7.17 ± 0.02	76^F^
	MF265	9.62 ± 0.06	7.30 ± 0.05	76^F^
	MF33	9.35 ± 0.03	6.46 ± 0.12	69^G^
	MF548	9.60 ± 0.00	6.43 ± 0.07	67^G^
	MF4	9.54 ± 0.02	5.23 ± 0.03	55^H^
	MF303	9.41 ± 0.03	5.02 ± 0.03	53^H^
	MF376	9.73 ± 0.00	4.54 ± 0.05	47^I^
	MF380	9.50 ± 0.05	4.37 ± 0.01	46^I^
	MF352	8.43 ± 0.05	3.77 ± 0.10	45^I^
	MF556	9.50 ± 0.18	4.25 ± 0.07	45^I^

*L. brevis *	MF105	9.06 ± 0.03	8.93 ± 0.18	99^A^
	MF314	9.23 ± 0.09	9.18 ± 0.01	99^A^
	MF494	9.52 ± 0.08	9.19 ± 0.17	97^AB^
	MF493	8.96 ± 0.11	8.50 ± 0.12	95^B^
	MF343	9.36 ± 0.15	8.81 ± 0.16	94^B^

*P. ethanolidurans *	MF50	9.63 ± 0.01	<1.00	—
	MF179	9.51 ± 0.04	<1.00	—
	MF180	9.48 ± 0.00	<1.00	—

Values are expressed in mean ± standard error.

^A–I^Values with different letters within a column indicate significant differences between LAB strains (*p* < 0.05).

**Table 3:** Survival of LAB strains in simulated gastric and intestinal conditions.

Species	Strain number	Initial counts (log cfu/mL)	Survival after 4 h in the pepsin-pH 2.5 solution		Survival after 6 h in the pancreatin-bile salt solution (pH 8.0)	
			(log cfu/mL)	%	(log cfu/mL)	%
*L. plantarum *	MF4	8.20 ± 0.02	8.18 ± 0.20	100^A^	<1.00	—
	MF213	8.10 ± 0.17	8.08 ± 0.18	100^A^	<1.00	—
	MF376	8.32 ± 0.01	8.30 ± 0.05	100^A^	<1.00	—
	MF305	8.30 ± 0.15	8.23 ± 0.03	99^A^	<1.00	—
	MF352	8.30 ± 0.01	6.78 ± 0.11	82^B^	4.11 ± 0.01	50^B^
	MF548	8.25 ± 0.02	6.78 ± 0.19	82^B^	<1.00	—
	MF143	8.34 ± 0.11	6.60 ± 0.03	79^CD^	<1.00	—
	MF239	8.26 ± 0.03	6.54 ± 0.14	79^CD^	<1.00	—
	MF265	8.32 ± 0.14	6.48 ± 0.09	78^DE^	<1.00	—
	MF303	8.26 ± 0.03	6.30 ± 0.02	76^F^	3.30 ± 0.06	40^C^
	MF205	8.31 ± 0.04	6.08 ± 0.07	73^G^	3.30 ± 0.11	40^C^
	MF380	8.22 ± 0.01	6.00 ± 0.01	73^G^	3.30 ± 0.07	40^C^
	MF556	8.24 ± 0.09	6.00 ± 0.16	73^G^	3.48 ± 0.04	42^C^
	MF178	8.29 ± 0.15	5.51 ± 0.19	66^H^	3.48 ± 0.17	42^C^
	MF377	8.30 ± 0.01	5.48 ± 0.05	66^H^	5.15 ± 0.02	62^A^
	MF169	8.30 ± 0.03	5.43 ± 0.08	65^H^	5.16 ± 0.05	62^A^
	MF33	8.24 ± 0.05	4.36 ± 0.15	53^I^	<1.00	—

*L. brevis *	MF314	8.34 ± 0.01	8.28 ± 0.04	99^A^	3.48 ± 0.16	42^C^
	MF494	8.20 ± 0.17	6.54 ± 0.07	80^C^	5.18 ± 0.13	63^A^
	MF493	8.27 ± 0.13	6.38 ± 0.08	77^EF^	<1.00	—
	MF105	8.31 ± 0.02	6.30 ± 0.03	76^F^	5.08 ± 0.01	61^A^
	MF343	8.29 ± 0.04	<1.00	—	5.07 ± 0.12	61^A^

*P. ethanolidurans *	MF50	8.26 ± 0.06	<1.00	—	<1.00	—
	MF179	8.22 ± 0.11	<1.00	—	<1.00	—
	MF180	8.34 ± 0.09	<1.00	—	<1.00	—

Values are expressed in mean ± standard error.

^A–I^Values with different letters within a column indicate significant differences between LAB strains (*p* < 0.05).

**Table 4:** Cholesterol assimilation and surface hydrophobicity properties of LAB strains.

Species	Strain number	Cholesterol assimilation^*∗*^ (%)	Surface hydrophobicity (%)
*L. plantarum *	MF4	11.72 ± 0.92^DEF^	2.39 ± 1.57^M^
	MF33	43.57 ± 1.37^B^	3.14 ± 1.16^LM^
	MF143	47.69 ± 0.37^A^	39.62 ± 1.20^G^
	MF169	44.26 ± 0.41^B^	1.42 ± 1.42^M^
	MF178	12.00 ± 2.47^DE^	32.7 ± 0.56^H^
	MF205	3.85 ± 0.82^I^	3.37 ± 1.92^LM^
	MF213	1.89 ± 0.87^I^	3.18 ± 2.27^LM^
	MF239	9.80 ± 1.65^EFG^	52.02 ± 0.58^E^
	MF265	2.30 ± 1.10^I^	82.41 ± 0.59^B^
	MF303	9.76 ± 0.78^EFG^	0.91 ± 0.27^M^
	MF305	14.06 ± 0.69^CD^	17.72 ± 0.10^I^
	MF352	9.89 ± 0.46^EFG^	8.07 ± 0.27^JK^
	MF376	4.27 ± 1.05^HI^	43.72 ± 0.49^F^
	MF377	8.11 ± 0.14^FG^	2.01 ± 0.77^M^
	MF380	7.33 ± 0.64^GH^	0.66 ± 0.22^M^
	MF548	1.57 ± 0.37^I^	6.25 ± 0.42^KL^
	MF556	48.56 ± 1.24^A^	9.98 ± 1.60^J^

*L. brevis *	MF105	16.62 ± 2.88^C^	97.96 ± 0.10^A^
	MF314	1.79 ± 0.14^I^	8.53 ± 1.24^JK^
	MF343	8.43 ± 0.82^EFG^	0.17 ± 0.17^M^
	MF493	0.83 ± 0.09^I^	62.36 ± 2.59^D^
	MF494	1.61 ± 0.05^I^	67.29 ± 0.16^C^

Values are expressed in mean ± standard error.

^A–M^Values with different letters within a column indicate significant differences between LAB strains (*p* < 0.05).

^*∗*^Total cholesterol concentration of the control broth was measured as 45.72 ± 0.46 *μ*g/mL.
